# Successful percutaneous transhepatic lymphangiography and embolization for intractable hepatic lymphorrhea after laparoscopic distal gastrectomy: a case report

**DOI:** 10.1186/s40792-023-01615-w

**Published:** 2023-02-27

**Authors:** Kohei Harigane, Hiroshi Nemoto, Yoshiyuki Yoshida, Hiromasa Komori, Hideki Sarukawa, Naoki Yazawa, Taku Miyamae

**Affiliations:** 1 Department of Surgery, Ebina General Hospital, 1320 Kawaraguchi, Ebina, 243-0433 Japan; 2 Department of Interventional Radiology, Ebina General Hospital, 1320 Kawaraguchi, Ebina, 243-0433 Japan

**Keywords:** Hepatic lymphorrhea, Laparoscopic distal gastrectomy, Percutaneous transhepatic lymphangiography, Embolization

## Abstract

**Background:**

Hepatic lymphorrhea is a rare and serious complication of surgery for digestive tract cancers and is thought to occur as a result of lymph node dissection of the hepatoduodenal ligament. This complication results in the accumulation of lymphatic fluid, which may in turn lead to nutritional disorders, immune deficiency, and circulation insufficiency. However, there is currently no standard strategy for treating this condition.

**Case presentation:**

A 49-year-old woman with alcoholic liver damage underwent laparoscopic distal gastrectomy with lymph node dissection for early gastric cancer. Abundant ascites persisted postoperatively, and the fluid was suspected to indicate hepatic lymphorrhea. The patient was re-admitted on postoperative day 26 due to the onset of a brain infarction caused by dehydration. Various conservative treatments for hepatic lymphorrhea were ineffective. She underwent percutaneous transhepatic lymphangiography and embolization on postoperative day 81, with obvious effect. Computed tomography images demonstrated complete disappearance of ascites.

**Conclusions:**

Postoperative hepatic lymphorrhea is a rare and serious complication of radical surgery for digestive tract cancers. The current case suggests that percutaneous transhepatic lymphangiography and embolization may be a rational treatment option when conservative treatments fail.

## Background

Hepatic lymphorrhea is a rare and serious complication of surgery for digestive cancers. It is considered to occur as a result of lymph node dissection of the hepatoduodenal ligament. This complication results in the accumulation of lymphatic fluid, which may in turn lead to nutritional disorders, immune deficiency, and circulation insufficiency. However, there is currently no standard strategy for treating this condition [1].

We experienced a patient who developed hepatic lymphorrhea following laparoscopic distal gastrectomy (LDG). Conservative medical management was not successful, but the patient’s condition improved after embolization with *n*-butyl-2-cyanoacylate (NBCA) under percutaneous transhepatic lymphangiography (PTL). This represents the first report in Japan of the use of this procedure to treat a patient with hepatic lymphorrhea after gastrectomy for gastric cancer.

## Case presentation

A 49-year-old woman underwent screening gastroendoscopy because of chronic liver damage, which detected an ulcerous lesion in the lesser curvature of her pylorus. Carcinoma was suspected macroscopically but biopsy results failed to confirm the diagnosis; however, gastrectomy was planned. The patient had no prior history of abdominal surgery. However, she was a heavy drinker, and the laboratory data revealed liver damage. She underwent LDG including lymph node incision using an ultrasonic coagulation cutting device. No lymphatic leakage was detected in the operation field, and a drainage tube was placed after completion of the Billroth-I reconstruction. The duration of the operation was 247 min and the total blood loss was 10 ml. Early gastric adenocarcinoma with radical resection was confirmed by histopathology.

**Fig. 1:**
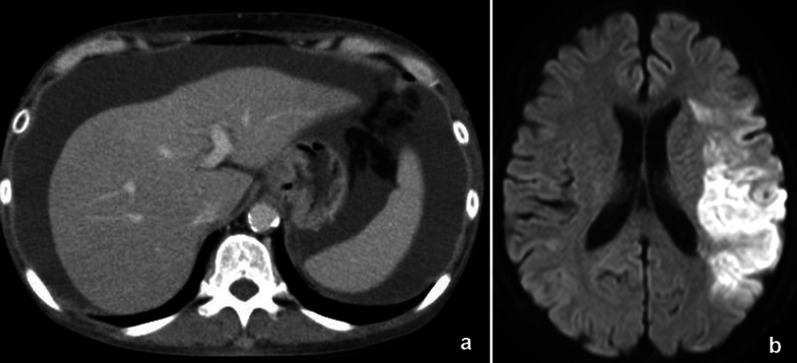
Imaging findings at onset of brain infarction. **a**. Enhanced computed tomography image showing abundant ascites; **b** left brain infarction presented by magnetic resonance imaging

Postoperatively, 200–300 ml of clear fluid was discharged every day, and the patient was diagnosed with hepatic lymphorrhea. However, the patient discharged herself on postoperative day 11, despite no reduction in the volume of ascites. She was re-admitted on postoperative day 26 because of the onset of right hemiplegia, and was diagnosed with an infarction in the left middle cerebral artery. We considered that this was likely to have been caused by reduced cerebral blood flow resulting from systemic dehydration due to hepatic lymphorrhea (Fig. 1).

The patient was treated with sufficient infusion and albumin transfusion and her condition gradually improved. Ultrasound-guided PTL was attempted on day 34 by a specialist in interventional radiology (IR) for hepatic lymphorrhea, but visualization of the lymph vessel was unsuccessful.

To prevent loss of ascites from a drainage tube, a peritoneovenous shunt (PVS) was placed under local anesthesia, and her activities of daily living recovered. The PVS tube was clamped temporarily on postoperative day 54 in preparation for ending the procedure, but her ascites increased again and the PVS was continued.

**Fig. 2:**
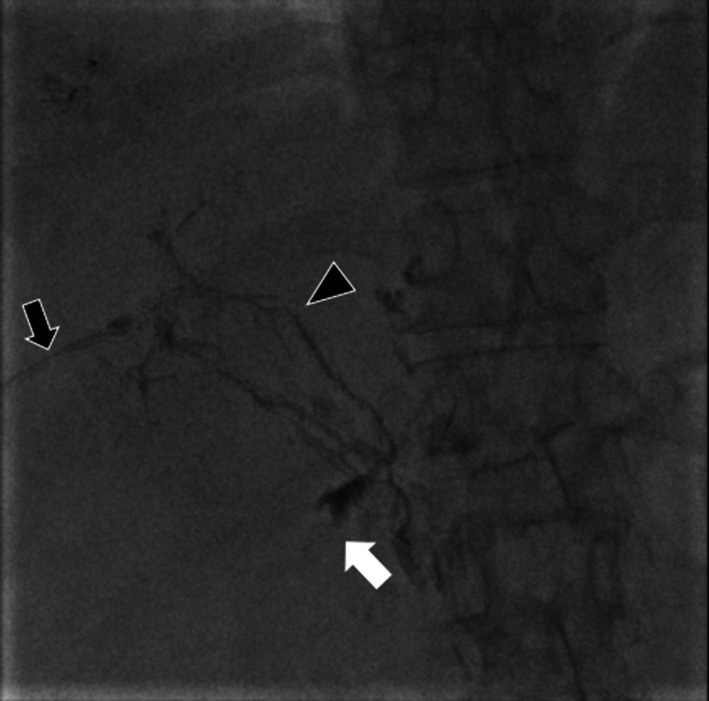
Findings of percutaneous transhepatic lymphangiography (PTL). PTL image showing lymph vessel (black arrowhead) and point of leakage (white arrow) identified by contrast medium injected using a 22G Chiba needle (black arrow)

On day 61, a 2nd PTL was planned to remove the shunt and perform Lipiodol embolization. We considered that the lack of success of the 1st procedure was attributable to the 1st PTL puncture being distal to the hepatic hilum and, therefore, made the puncture for the 2nd PTL proximal to the hepatic hilum. A 22G Chiba needle was inserted by an IR specialist, from the right hypochondrium to the junction of the right superior and right anterior branches of the portal vein. The space of Disse was then delineated using a water-soluble contrast medium. The contrast medium flowed downstream, and lymphatic leakage was confirmed at the site of the hepatoduodenal ligament (Fig. 2). Lipiodol (oily contrast medium) 4 ml was then injected and computed tomography images showed all the contrast agent located in the duodenal ligament, suggesting that the procedure had been effective. However, her abdominal distention improved temporarily but then worsened again 3 weeks later. Given that a lymph vessel had developed, we considered that the short-term effectiveness of the Lipiodol injection was attributable to incomplete embolism of the point of leakage.

**Fig. 3:**
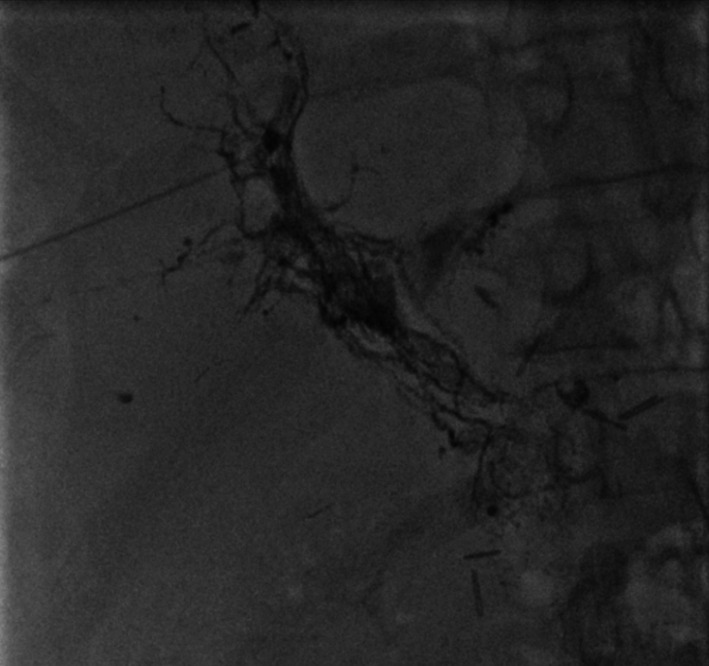
Computed tomography after percutaneous transhepatic lymphangiography and embolization. Image showing accumulation of Lipiodol and *n*-butyl-2-cyanoacylate from the hepatic hilum to the hepatic lymphatic vessels (circle) and disappearance of ascites

**Fig. 4:**
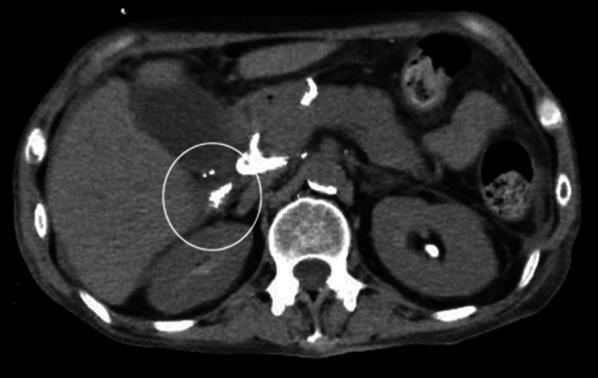
Findings of leakage disappeared in the imaging after PTLE

PTL on day 81 showed a remarkably developed lymphatic vessel. A mixture of Lipiodol and NBCA 0.5 ml was then injected to embolize the lymph vessel with obvious results (Fig. 3). The patient’s ascites had disappeared by day 106 (Fig. 4), her condition, including her paralysis, improved, and she was discharged on postoperative day 108. The only complications of the percutaneous transhepatic lymphangiography and embolization (PTLE) were minor abdominal pain and a slight worsening of hepatic function. One year after gastric resection, the patient remained disease-free without paralysis.

## Discussion

The liver produces 25–50% of the thoracic–duct lymph, and lymphatic damage caused by dissection of the lymph node in the gastroduodenal ligament may cause hepatic lymphorrhea [2]. Liver damage causes an increase in portal vein pressure, a reduction in portal vein blood flow, impairment of the permeability of the blood–lymph barrier, and development of a dense portal lymphatic network, and is considered to result in an increase in liver lymph flow [3]. Although most lymphatic ductal injuries are temporary and recover by tissue regeneration, increasing lymphatic flow and prevention of recovery may cause hepatic lymphorrhea in patients with chronic liver damage [4], with a high mortality rate in cirrhotic patients who develop this complication after surgery [5]. In addition, as in the current case, liver damage is considered to a contributory factor. This complication could possibly be prevented using clips, as well as a sealing device, during lymph node dissection.

Twenty-six cases of hepatic lymphorrhea after gastrectomy for gastric cancer have been reported in Japanese or English, including the current case (Table 1) [4, 6–26]. The average patient age was 54.9 (range 32–79) years, 73% of patients were male, 69% had various forms of liver damage, 54% were operated on for early cancer, and 23% underwent total gastrectomy. All cases of hepatic lymphorrhea were initially treated by conservative management, such as total parenteral nutrition and transfusion, followed by various invasive procedures. The main invasive treatments were sclerotherapy with OK-432 (50%), ligation of the damaged vessel (38%), PTLE (15%), and other procedures.

**Table 1:** Reported cases of hepatic lymphorrhea after gastrectomy for gastric cancer (published in Japanese or English)

Author	Year	Age (years)	Sex	Liver disease	Type of GC	Surgery	Conservative treatment	Invasive treatment				
								Reoperation		OK-432 sclerotherapy (no. injections)	PTL	
								Surgical ligation	Other			PTLE
Miyagawa et al. [6]	1983	65	M	CH,HBV	AC	TG	●	●				
Nakashima et al. [7]	1985	58	M	none	AC	DG	●	●	●			
Nakano et al. [8]	1987	49	M	none	AC	TG	●	●	●			
Kawata et al. [9]	1989	52	M	LC	EC	DG	●		●			
Umehara et al. [10]	1989	59	F	none	AC	TG	●	●				
Kaneko et al. [11]	1991	44	F	none	EC	DG	●	●				
Shimizu et al. [12]	1992	62	M	LC, HCV	EC	DG	●	●		3		
Ota et al. [13]	1993	70	M	CH, HCV	EC	DG	●	●	●			
Tajima et al. [14]	1993	59	M	none	EC	DG	●		●			
Sasaki et al. [15]	1994	54	M	LC	EC	DG	●			5		
Kawahira et al. [16]	1994	58	F	CH, HCV	EC	DG	●		●	1		
Matsumoto et al. [17]	1995	44	F	CH, HBV	AC	DG	●	●				
Tada et al. [18]	1996	79	M	CH, HCV	EC	DG	●			6		
Tada et al. [18]	1996	57	F	CH, HCV	EC	DG	●			3	●	
Takahata et al. [19]	1998	49	M	CH	AC	DG	●	●	●	1		
Yoshida et al. [20]	2000	55	M	LC, HBV	AC	DG	●			2		
Matsumoto et al. [21]	2000	57	F	ND	EC	PR	●			1	●	
Ogasawara et al. [22]	2002	63	M	CH, HCV	AC	TG	●			5		
Tatsuzawa et al. [23]	2002	54	M	CH, HCV	EC	DG	●			5		
Isogai et al. [24]	2002	50	M	CH, alcoholic	EC	DG	●			3		
Inaba et al. [4]	2003	49	M	CH, HCV	AC	DG	●			5		
Tanaka et al. [25]	2004	66	M	CH, HBV	EC	TG	●	●	●	6		
Nguyen et al. [26]	2020	32	M	ND	ND	PR	●				●	●
Nguyen et al. [26]	2020	56	M	ND	ND	TG	●				●	●
Nguyen et al. [26]	2020	37	M	ND	ND	PR	●				●	●
Current case	2022	49	F	CH, alcoholic	EC	LDG	●				●	●

*GC* gastric cancer; *PTL* percutaneous transhepatic lymphangiography; *PTLE* percutaneous transhepatic lymphangiography and embolization; *No.* number; *Other* other procedure; *M* male; *F* female; *CH* chronic hepatitis; *HBV* hepatitis B virus; *HCV* hepatitis C virus; *AC* advanced cancer; *EC* early cancer; *ND* no description; *DG* distal gastrectomy; *TG* total gastrectomy; *PR* partial resection; *LDG* laparoscopic distal gastrectomy

Lymph vessel ligation requires an invasive re-operation, which is complicated by postoperative adhesiolysis and difficulties identifying the injury site. Although some ligation procedures were successful [6, 10], the perforation site could not be identified in some cases [14, 19] and no effect of ligation was detected after re-operation in others [17].

Although the intraperitoneal injection of OK-432 is a relatively easy method that can be carried out under local anesthesia, the agent may be diluted in the large space, and there are concerns about potential complications, such as systemic fever, abdominal pain, and adhesive ileus. An average of 3.5 (1–6) injections were required for recovery, with five or more injections needed in about half of all cases [4, 15, 18, 22, 23, 25].

Tada et al. identified hepatic lymphorrhea using PTL in 1996 [18]. Even PTL without embolization can cause inflammation of the lymphatic endothelium and perivessel tissue, and can, therefore, effectively prevent leakage in some cases [21, 27]. PTLE involves embolism of the lymph vessel by adding an embolic agent, such as NBCA, to the contrast medium. The mixture of NBCA and Lipiodol can then stop blood vessel hemorrhage by damaging the endothelium, and may also close perforated sites in the lymphatic duct by the same mechanism [28].

There have only been 10 reported cases of the use of PTLE to resolve postoperative hepatic lymphorrhea following surgery for digestive cancer [26, 27, 29–31], and the current case is the 4th case in a patient with gastric cancer (Table 2). All these cases recovered clinically after the 1st or 2nd embolization, suggesting that the method is very effective. However, the lymph vessel could not be delineated at the 1st PTL in four cases, including the current case [26, 27, 30].

**Table 2:** Successful cases treated by percutaneous transhepatic lymphangiography and embolization of hepatic lymphorrhea after surgery for digestive cancer

Author	Year	Sex	Age (years)	Cancer	Surgery	No. of PTLE
Guez et al. [29]	2014	M	56	PC	PD	1
Nguyen et al. [26]	2020	M	32	GC	PR	1
Nguyen et al. [26]	2020	M	56	GC	TG	1
Nguyen et al. [26]	2020	M	37	GC	PR	2
Nguyen et al. [27]	2021	M	59	PC	PD	2
Nguyen et al. [27]	2021	M	59	PC	PD	1
Nguyen et al. [27]	2021	F	73	PC	PD	1
Hasegawa et al. [30]	2021	M	62	IPMN	PD	1
Dung et al. [31]	2022	M	49	HCC	Hepatectomy	1
Current case	2022	F	49	GC	LDG	1

*PTLE* percutaneous transhepatic lymphangiography and embolization; *No.* number; *M* male; *F* female; *PC* pancreatic cancer; *GC* gastric cancer; *IPMN* intraductal papillary mucinous neoplasm; *HCC* hepatocellular carcinoma; *PD* pancreatoduodenectomy

Potential complications of PTLE include hepatic dysfunction [26], but there have only been two reports of mild abdominal pain [30] and slight intraabdominal hemorrhage [27] to date. Although there is a concern about possible embolism in other organs, there have been no reports of this occurring after PTLE for hepatic lymphorrhea after surgery for a digestive tract cancer. In one study, only one case of asymptomatic pulmonary embolism occurred among 106 patients who had undergone lymphangiography and embolization for postoperative lymphorrhea [2]. Sclerotherapy with OK-432 and PTLE are effective procedures that can be performed under local anesthesia. However, fewer PTLE compared with sclerotherapy procedures may be required in patients who fail to respond to conservative treatment. PTLE may be a best treatment option for postoperative hepatic lymphorrhea if a skilled interventional radiologist is available at the institution.

## Conclusions

Postoperative hepatic lymphorrhea is a rare and serious complications of radical surgery for digestive tract cancers. The current case suggests that PTLE may be a rational treatment option when conservative treatments fail.

## Abbreviations

IR: Interventional radiology
LDG: Laparoscopic distal gastrectomy
NBCA: *N*-Butyl-2-cyanoacylate
PTL: Percutaneous transhepatic lymphangiography
PTLE: Percutaneous transhepatic lymphangiography and embolization
PVS: Peritoneovenous shunt

## References

[1] Sommer CM, Pleper CC, Offensperger F, Pan F, Killguss HJ, Konlnger J (2021). Radiological management of postoperative lymphorrhea. Langenbecks Arch Surg.

[2] Inoue M, Nakamatsu S, Yashiro H, Tamura M, Suyama Y, Tsukada J (2016). Lymphatec intervention for various type of lymphorrhea: access and treatment. Radiographics.

[3] Ohtani O, Ohtani Y (2008). Lymph circulation in the liver. Anat Rec.

[4] Inaba Y, Arai Y, Matsuda K, Aramaki T, Kodera Y (2003). Intractable massive ascites following radical gastrectomy, treatment with local intraperitoneal administration of OK-432 using unified CT and fluoroscopy system. Australas Radiol.

[5] Isozaki H, Okajima K, Ichinona T, Fujii K, Nomura E, Izumi N (1997). Surgery for gastric cancer in patients with cirrhosis. Surg Today.

[6] Miyagawa S, Tokio Y, Kawahara H, Nakajima H, Nishigki K, Sasago Y (1983). A case of intractable lymphatic ascites after gastrectomy for gastric cancer (in Japanese). Geka Shinryo.

[7] Nakashima R, Fujita T, Shirasaki K, Hokari I, Karaki Y, Fujimaki M (1985). Severe hepatic ascites following distal subtotal gastrectomy; report of a case (in Japanese). Rinsho Geka.

[8] Nakano T, Sato Y, Matsuri K, Miyata G, Sano J, Tomita S (1987). Operated case of intractable ascites after radical gastrectomy for gastric cancer (in Japanese). Iwate Kenritsu Ikadaigak Zassi.

[9] Kawata N, Morita H, Matsumoto K, Iwahashi K, Tsunekawa K (1989). A case of hepatic lymphorrhea after radical gastric cancer operation with liver cirrhosis (in Japanese with English abstract). Rinpa Gaku.

[10] Umehara Y, Miyahara T, Yoshida M, Oba N, Gotou H, Harada Y (1989). A case report of intractable hepatic lymphorrhea following radical gastrectomy for gastric cancer (in Japanese). Jpn J Gastroenterol Surg.

[11] Kaneko Y, Tanaka M, Hayashi H, Ietsugu K, Iwagami S, Kiyokawa H (1991). A case report of intractable massive ascites (hepatic lymphorrhea) following radical gastrectomy for early gastric cancer (in Japanese). Shokaki Geka.

[12] Shimizu Y, Tanaka T, Nakae A, Itoi H, Matui S, Nagasima K (1992). A case of intractable massive ascites (hepatic lymphorrhea) following radical gastrectomy for early gastric cancer (in Japanese with English abstract). Kyoto Furitsu Ika Daigaku Zasshi.

[13] Ota H, Miyazawa T, Inaba H, Ueda N, Maeura Y, Matsunaga S (1993). A case report of intractable ascites due to hepatic lymphorrhea from hepatoduodenal ligament after radical gastrectomy for gastric cancer (in Japanese with English abstract). Nihon Shokaki Geka Gakkai Zassi.

[14] Tajima S, Mitui M, Sato D, Monmyo U, Ogawa M (1993). Operated case of hepatic lymphorrhea after operation for gastric cancer (in Japanese with English abstract). Nihon Rinsho Geka.

[15] Sasaki H, Nakagawa K, Siki S, Yamashita Y, Yumura M, Kodani J (1994). An effective treatment by intraperitoneal OK-432 for intractable hepatic lymphorrhea after radical gastrectomy for early gastric cancer with liver cirrhosis. A case report (in Japanese with English abstract). Nihon RinshoGgeka Igakukai Zasshi.

[16] Kawahira Y, Nakao K, Nakahara M, Hamaji M, Ogino N, Miyazaki S (1994). A case of intractable hepatic lymphorrhea after gastrectomy for gastric cancer (in Japanese with English abstract). Jpn J Gastroenterol Surg.

[17] Matsumoto H, Nomura S, Washio K, Fujioka M, Kobashi Y, Usui Y (1995). A case of intractable hepatic lymphorrhea following radial gastrectomy for a gastric cancer (in Japanese with English abstract). Nihon RinshoGgeka Igakukai Zasshi.

[18] Tada I, Arita T, Abe T, Yasuda K, Kano T, Kaketani K (1996). Intractable massive ascites following radical gastrectomy for an early gastric cancer-two case reports (in Japanese with English abstract). Nihon Rinshogeka Gakagakkai Zasshi.

[19] Takahata H, Obino K, Nakano H (1998). A case of intractable hepatic lymphorrhea following radical gastrectomy for gastric cancer (in Japanese). Rinsho Geka.

[20] Yoshida M, Matsuyama H (2000). A case of an effective treatment with intraperitoneal OK-432 administration for intractable hepatic lymphorrhea after radical gastrectomy for gastric cancer with liver cirrhosis (in Japanese with English abstract). Tokyo Joshi Ikadaigaku Zassi.

[21] Matsumoto S, Mori H, Tada I (2000). Successful demonstration of post-operative lymphatic fistula by percutaneous transhepatic lymphography. Clin Radiol.

[22] Ogasawara Y, Higashi K, Okano K (2002). A successful conservative therapy with intraperitoneal OK-432 administration for intractable hepatic lymphorrhea following a radical gastrectomy for gastric cancer. A case report (in Japanese with English abstract). Nihon Rinshogeka Gakkaizasshi.

[23] Tatsuzawa Y, Nozaki, Kinoshita T, Shimizu J, Kawaura Y (2002). Effective treatment by intraperitoneal OK-432 for intractable hepatic lymphorrhea gastrectomy for gastric cancer (in Japanese). Rinsho Geka.

[24] Isogai M, Yamaguchi A, Harada T, Kaneoka Y, Suzuki M, Akutagawa A (2002). A case of intractable hepatic lymphorrhea after operation for gastric cancer treated successfully by intraperitoneal administration of OK-432 (in Japanese). Shokaki Geka.

[25] Tanaka K, Ohmori Y, Mohri Y, Tonoguchi H, Suematsu M, Taguchi Y (2004). Successful treatment of refractory hepatic lymphorrhea after gastrectomy for early gastric cancer, using surgical ligation and subsequent OK-432 (Picibanil) sclerotherapy. Gastric Cancer.

[26] Nguyen NC, Inoue M, Le TL, Pham HC, Trinh HS, Pham DH (2020). Intrahepatic lymphatic channel sclerotic embolization for treatment of postoperative lymphatic ascites: a report of 3 cases. Radiol Case Rep.

[27] Nguyen TK, Luong TH, Nguyen NC, Nguyen HH, Le VK, Trinh HS (2021). Hepatic lymphorrhea following pancreaticoduodenectomy: preliminary diagnosis and treatment experience from case series of four patients. Ann Med Surg (Lond).

[28] Hur S, Shin JH, Lee IJ, Min S-K, Min S-I, Ahn S (2016). Early experience in management of postoperative lymphatic leakage using lipiodol lymphangiography and adjunctive glue embolization. J Vasc Interv Radiol.

[29] Guez D, Nadolski GJ, Pukenas BA, Itkin M (2014). Transhepatic lymphatic embolization of intractable hepatic lymphoma. J Vasc Interv Radiol.

[30] Hasegawa T, Tsuboi M, Fukushima K, Yukawa T (2021). Refractory hepatic lymphorrhea: percutaneous transhepatic lymphangiography and embolization with n-butyl-2-cyanoacrylate glue. Cardiovasc Intervent Radiol.

[31] Dung LV, Binh NT, Linh LT, Hien PN, Dung TN, Long TB (2022). Percutaneous embolization of hepatic lymphorrhea post-hepatectomy. Radiol Case Rep.

